# 注射用两性霉素B胆固醇硫酸酯复合物治疗血液系统恶性肿瘤合并侵袭性真菌病30例临床分析

**DOI:** 10.3760/cma.j.issn.0253-2727.2022.10.008

**Published:** 2022-10

**Authors:** 俊 王, 松 金, 小津 吴, 瞄 苗, 晓文 唐, 雪峰 何, 惠英 仇, 悦 韩, 荧 王, 渭阳 李, 彩霞 李, 胜利 薛, 骁 马, 德沛 吴

**Affiliations:** 国家血液系统疾病临床医学研究中心，江苏省血液研究所，苏州大学附属第一医院血液科，苏州大学造血干细胞移植研究所，苏州 215006 National Clinical Research Center for Blood Diseases, Jiangsu Institute of Hematology, the First Affiliated Hospital of Soochow University, Institute of Blood and Marrow Transplantation, Soochow University, Jiangsu 215006, China

**Keywords:** 侵袭性真菌病, 两性霉素B胆固醇硫酸酯复合物, 经验治疗, 诊断驱动治疗, Invasive fungal disease, Amphotericin B cholesteryl sulfate complex, Empirical antifungal therapy, Diagnostic-driven antifungal therapy

## Abstract

**目的:**

评价注射用两性霉素B胆固醇硫酸酯复合物（ABCD）经验治疗和诊断驱动治疗血液系统恶性肿瘤合并侵袭性真菌病患者的安全性和有效性。

**方法:**

纳入30例2021年6月至2021年8月在苏州大学附属第一医院与苏州弘慈血液病医院接受ABCD经验治疗和诊断驱动治疗的血液系统恶性肿瘤合并侵袭性真菌病患者。主要研究终点为安全性。次要研究终点包括治疗有效率、发热持续时间和ABCD治疗完成率。

**结果:**

共纳入30例患者，24例（80.0％）完成了ABCD治疗。不良反应多为1～2级，其中输液反应（24/30，80.0％）为最常见的不良反应。总体治疗有效率为80.0％（24/30）。全部患者中，24例（80.0％）发热持续时间为1 d。

**结论:**

ABCD治疗血液系统恶性肿瘤合并侵袭性真菌病具有较好的疗效及安全性。

## Abstract (en)

**Objective:**

To assess the safety and effectiveness of amphotericin B cholesteryl sulfate complex for injection in the context of empirical and diagnostic antifungal therapy for patients with hematological malignancies in addition to invasive fungal illness.

**Methods:**

This single-arm clinical study enrolled 30 patients who received empirical and diagnostic-driven antifungal therapy for hematological malignancies combined with invasive fungal disease. The primary endpoint was safety. Response rate, fever duration, and treatment completion rate were all considered secondary objectives.

**Results:**

30 participants were eventually enrolled in the study，and the treatment completion rate was 80.0％. Most adverse events were in grades 1–2. Infusion response was the most frequent adverse event（24/30, 80％）. The overall response rate was 80.0％（24/30）. In 24 patients（80.0％）, the fever persisted for 1 day.

**Conclusion:**

Treatment of invasive fungal illness in conjunction with hematological malignancies showed good efficacy and safety with amphotericin B cholesteryl sulfate complex for injection.

侵袭性真菌病（IFD）系指真菌侵入人体，在组织、器官或血液中生长、繁殖，并导致炎症反应及组织损伤的感染性疾病[1]。近年来，IFD的发病率呈持续上升趋势[2]–[3]，是血液系统恶性肿瘤患者死亡的危险因素[4]–[5]。目前，治疗真菌感染的药物主要包括三唑类、多烯类和棘白菌素类。国内多烯类药物包括两性霉素B、两性霉素B脂质体和两性霉素B胆固醇硫酸酯复合物（ABCD），其活性物质均为两性霉素B。三唑类和棘白菌素类药物耐药趋势日益上升，但两性霉素B的耐药情况鲜有报道[6]。肾毒性是两性霉素B的主要剂量限制性毒性，而两性霉素B含脂制剂可有效降低毒性、提高安全性[7]–[10]。ABCD是两性霉素B与胆固醇酰硫酸酯钠按1∶1的分子摩尔比形成的盘状纳米粒子胶体分散体，本研究我们将注射用ABCD经验治疗和诊断驱动治疗在血液系统恶性肿瘤合并IFD患者中的安全性和有效性报道如下。

## 病例与方法

1. 病例：纳入2021年6月至2021年8月就诊于苏州大学附属第一医院与苏州弘慈血液病医院的30例血液系统恶性肿瘤合并IFD患者。纳入标准：①年龄14～70岁；②血液系统恶性肿瘤患者且接受化疗或造血干细胞移植（HSCT）；③筛选前48 h内，至少2次（间隔30 min以上）口腔温度≥38.3 °C（腋下温度≥38.0 °C）或≥38.0 °C（腋下温度≥37.7 °C）持续1 h以上；④既往接受至少96 h的广谱抗菌治疗但仍出现复发性或持续性发热且怀疑真菌感染；⑤预期生存时间≥3个月。排除标准：①两性霉素B类药物或胆固醇硫酸酯复合物过敏；②筛选前1个月内曾接受2种及以上的抗真菌药物治疗；③肝功能异常［天冬氨酸转氨酶（AST）或丙氨酸转氨酶（ALT）≥ 5倍正常值上限（ULN）、或ALT或AST≥3倍ULN且总胆红素≥1.5倍ULN］；④肾功能减退需要或者目前正在进行血液透析或腹膜透析；⑤临床上有意义的低钾血症（血清钾浓度<3.2 mmol/L或正在接受洋地黄化治疗血钾低于正常值下限）且开始试验治疗前低钾血症不能纠正；⑥孕妇、哺乳期妇女；⑦其他研究者认为不宜参加临床试验的情况。本研究获得苏州大学附属第一医院医学伦理委员会批准［批件号：2021伦审批第（106）号］，临床试验注册号：ChiCTR2100049329。所有研究对象均知情同意并签署知情同意书。

2. 治疗方案：研究药物为注射用ABCD，由石药集团欧意药业有限公司提供，规格为50 mg/瓶。采用地塞米松（2.0～5.0 mg静脉推注）±苯海拉明预防输液反应。将少量研究药物（10 ml稀释液含有1.6～8.3 mg）滴注15～30 min进行测试并观察30 min，能耐受者接受后续治疗。ABCD的初始剂量为0.5 mg·kg^−1^·d^−1^，第2天1.0 mg·kg^−1^·d^−1^，第3天增加至治疗剂量，其中接受造血干细胞移植患者的治疗剂量为1.0～4.0 mg·kg^−1^·d^−1^；接受化疗的患者治疗剂量为3.0～4.0 mg·kg^−1^·d^−1^。治疗周期为14 d。研究者根据临床实际调整剂量。按照下列公式计算每天给药的相对剂量强度（第2天后的计划给药剂量以3.0 mg·kg^−1^·d^−1^计），并根据给药总天数计算研究期间的平均相对剂量强度。

$$相对剂量强度(\text{\%})=\frac{实际给药剂量}{计划给药剂量}\times 100\text{\%}$$

3. 疗效评价：包括治疗有效率[11]、发热持续时间、ABCD治疗完成率。

4. 安全性评价：观察治疗期间与研究药物相关不良反应（输液反应、肾毒性、低钾血症及肝功能异常等）的发生率及严重程度。肾毒性定义为血清肌酐水平较基线水平增加一倍，或计算的肌酐清除率[12]下降超过50％。不良反应根据美国国家癌症研究所常见不良反应评价标准5.0版进行分级。

5. 统计学处理：应用SPSS 25.0软件进行统计分析。计量资料采用均值±标准差或中位数（范围）进行描述。计数资料采用例数及百分比进行描述。采用Pearson法进行相关性分析。*P*<0.05为差异有统计学意义。

## 结果

一、一般资料

2021年6月至2021年8月间共纳入30例患者，中位年龄39.5（16～69）岁，男20例（66.7％），女10例（33.3％），中位体重65（36～103）kg。疾病类型：急性髓系白血病18例（60.0％）、骨髓增生异常综合征7例（23.3％）、急性淋巴细胞白血病3例（10.0％）、T淋巴母细胞白血病/淋巴瘤2例（6.7％）。8例患者既往接受HSCT，22例患者既往仅接受诱导和（或）巩固化疗。感染部位：肺部感染15例（50.0％）、肝脏感染1例（3.3％）、脾脏感染1例（3.3％），接受诊断驱动治疗；13例（43.3％）无明确感染部位（未进行影像学相关检查或检查结果未提示感染），接受经验治疗。29例患者既往接受其他抗真菌治疗，其中伏立康唑17例（56.7％）、卡泊芬净8例（26.7％）、米卡芬净3例（10.0％）、泊沙康唑1例（3.3％）。

二、治疗

1. 剂量爬坡：30例患者中27例完成剂量爬坡，3例仅用药1～2 d，未完成剂量爬坡。8例接受HSCT的患者中，1例无维持剂量，余7例平均维持剂量为（1.9±0.9）mg·kg^−1^·d^−1^；22例接受化疗患者中，2例无维持剂量，余20例平均维持剂量为（2.2±0.7）mg·kg^−1^·d^−1^。

2. 药物暴露：8例接受HSCT患者中，仅1例在第2、3天未达到计划治疗剂量。22例接受化疗患者中，11例平均相对剂量强度>80％，6例平均相对剂量强度为60％～80％，5例平均相对剂量强度为40％～<60％。

3. ABCD治疗完成率：24例（80.0％）患者完成治疗，其中23例（76.7％）完成方案要求的14 d给药，1例（3.3％）用药11 d治疗有效后出院。6例（20.0％）患者未完成治疗，其中4例（13.3％）因寒战停药，1例（3.3％）因低血压停药，1例（3.3％）用药11 d后因死亡退出研究。

三、安全性评价

治疗过程中发生的不良反应详见表1。总体不良反应发生率为96.7％（29/30）。发生率≥5％的不良反应为输液反应［24/30，80.0％，包括发热（19/30，63.3％）及寒战（15/30，50.0％）］、血肌酐升高（5/30，16.7％）、低镁血症（3/30，10.0％）、低血压（3/30，10％）、高血压（3/30，10.0％）、皮疹（2/30，6.7％）及低钾血症（2/30，6.7％），多为1～2级（表1）。根据目标药物治疗期间的血肌酐评估患者肾功能，5例（16.7％）治疗后有血肌酐增高事件，其中2例在治疗结束后第7天随访时恢复至基线水平，3例在治疗结束后第14天随访时恢复至基线水平。5例血肌酐水平升高患者中2例（6.7％）在治疗过程中发生肾毒性事件，其中1例治疗过程中血肌酐水平较基线水平增加101.4％，计算的肌酐清除率下降56.7％，另1例血肌酐水平较基线水平增加86.1％，计算的肌酐清除率下降51.6％（图1），无肝功能异常事件发生。5例患者因寒颤（4例）及低血压（1例）退出研究，可能与研究药物有关。1例患者因T淋巴母细胞白血病/淋巴瘤、感染、肺出血与呼吸循环衰竭导致死亡，经临床判断与研究药物无关。相关性分析显示，血肌酐水平与ABCD给药量无明显线性相关性（*r*＝−0.095，*P*＝0.624）（图2）。

**表1:** 两性霉素B胆固醇硫酸酯复合物治疗血液系统恶性肿瘤合并侵袭性真菌病中发生率≥5％的不良反应［例（％）］

不良反应	总体	Ⅰ级	Ⅱ级	Ⅲ级	Ⅳ级
输液反应					
发热	19（63.3）	13（43.3）	5（16.7）	1（3.3）	0（0）
寒战	15（50.0）	8（26.7）	5（16.7）	2（6.7）	0（0）
实验室监测异常					
血肌酐升高	5（16.7）	0（0）	5（16.7）	0（0）	0（0）
低镁血症	3（10.0）	2（6.7）	0（0）	1（3.3）	0（0）
低钾血症	2（6.7）	1（3.3）	1（3.3）	0（0）	0（0）
心血管系统					
低血压	3（10.0）	1（3.3）	0（0）	2（6.7）	0（0）
高血压	3（10.0）	1（3.3）	1（3.3）	1（3.3）	0（0）
皮肤及其他					
皮疹	2（6.7）	1（3.3）	1（3.3）	0（0）	0（0）

**图1:**
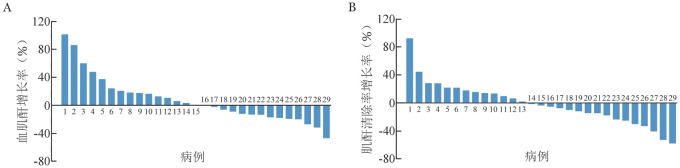
两性霉素B胆固醇硫酸酯复合物治疗29例血液系统恶性肿瘤合并侵袭性真菌病患者前后血肌酐（A）及肌酐清除率（B）较基线增长率（除外1例治疗1 d停止用药患者）

**图2:**
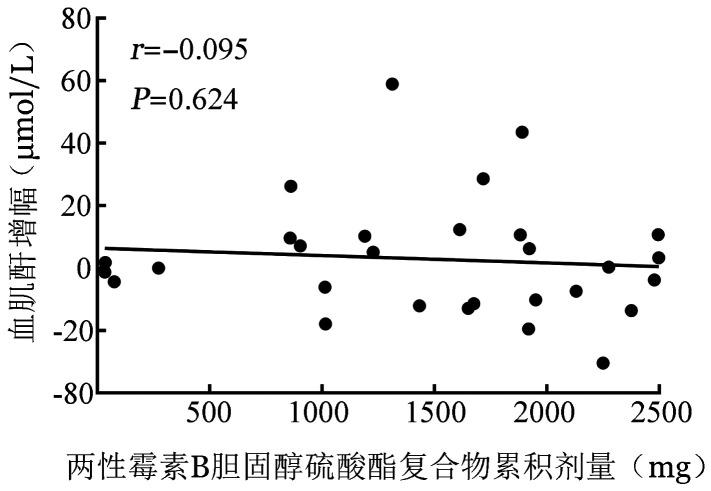
血肌酐水平较基线增幅与两性霉素B胆固醇硫酸酯复合物累积剂量的相关性分析（除外1例治疗1 d停止用药患者）

四、有效性评价

1. 治疗有效率：24例（80.0％）患者治疗有效，6例（20.0％）患者因不良事件（4例寒战和1例低血压）及死亡（1例）提前停药评估为治疗失败。总体治疗有效率为80.0％（24/30），接受经验治疗患者有效率为76.9％（10/13），诊断驱动治疗患者为82.4％（14/17）。HSCT患者治疗有效率为75.0％（6/8），化疗患者有效率为81.8％（18/22）。肺部感染治疗有效率为80.0％（12/15）、肝脏感染100％有效（1/1）、脾脏感染100％有效（1/1）、无明确感染部位76.9％有效（10/13）。

2. 发热持续时间：所有患者中，24例（80.0％）发热持续时间为1 d，4例（13.3％）发热持续时间为3 d，中位发热持续时间为1（1～8）d。经验性治疗患者中，中位发热持续时间为1（1～4）d，其中10例（76.9％）发热持续1 d；诊断驱动治疗患者中，中位发热持续时间为1（1～8）d，其中14例（82.4％）患者发热持续1 d。提示大部分患者的发热在1 d内得到缓解。

## 讨论

尽管两性霉素B已成为治疗全身真菌感染的经典药物，但其使用受到肾毒性及低钾血症的限制。一项两性霉素B治疗发热性中性粒细胞减少症的研究中，肾毒性发生率为41.1％，低钾血症发生率高达64.3％[13]。ABCD的安全性已在5个开放实验室Ⅰ/Ⅱ期临床试验中进行了测试[14]，选取了572例继发于严重基础疾病的真菌感染患者，ABCD剂量为6 mg·kg^−1^·d^−1^，在既往有肾衰竭的患者中，血肌酐水平没有差异，无肾毒性风险；低钾血症发生率仅8.0％，且对肝功能无显著影响；除血小板减少外，其他血常规指标与血生化参数没有显著改变；70例（12.2％）患者发生了与ABCD有关的不良事件，最常见的是输液相关不良事件。White等[15]开展的ABCD对比两性霉素B的对照研究中，共入组213例患者，结果表明ABCD组的肾功能损伤明显较少，输液反应较为常见。药代动力学研究显示国产ABCD与原研药药时曲线基本相近，曲线下面积高度吻合，其与原研药生物等效及组织分布一致，不良反应与原研药类似。

本组5例（16.7％）患者接受861～1 922 mg ABCD治疗后有血肌酐增高事件，但均在治疗结束后2周内恢复至正常水平。2例（6.6％）患者达到肾毒性标准，可能是联合使用多种抗生素和解热镇痛药物导致，但这2例患者治疗后的肌酐值仍在正常范围内（44.0～120.0 µmol/L），无实际临床意义。结果表明，使用ABCD治疗的患者肾功能较好，与报道ABCD能有效降低肾毒性一致[9]。此外，本研究表明血肌酐水平变化与ABCD累积剂量之间无明显线性相关性。这一结果表明与传统的两性霉素B[16]不同，ABCD与剂量依赖性肾毒性无关。三项关于ABCD的研究显示，ABCD治疗IFD的低钾血症发生率为5.0％～14.8％[8],[17]–[18]，本研究显示ABCD治疗的低钾血症不良事件发生率为6.7％，与既往研究基本一致。此外，本研究ABCD治疗期间出现与输液反应相关的不良事件，发热（12/19，63.2％）及寒战（13/15，86.7％）主要发生在给药的第1～2天，之后很少发生，这一结果与既往报道ABCD输液反应随着时间的推移而减少的规律相符，表明患者对ABCD耐受性的提高[19]–[20]。据报道，两性霉素B、两性霉素B脂质体、两性霉素B脂质复合体也存在类似不良反应[9],[21]。

本研究显示，ABCD具有较好疗效，有效率达80.0％，与既往研究报道ABCD治疗中国人群中IFD有效率（67.9％～96.7％）的结果一致[17],[22]–[23]。发热是真菌感染的重要临床指征，因此本研究还关注了患者治疗后的发热缓解情况。通过分析，显示80.0％的患者发热持续时间为1 d，由此可知在研究药物治疗较短时间内发热可得到缓解。不良事件除输液反应（寒战、发热）及血肌酐增高外，其他不良事件发生率均≤10％，且多为1～2级。

综上所述，ABCD治疗血液系统恶性肿瘤合并IFD具有较好的疗效及安全性。
